# Supplementary figures and images for: ClpP regulates breast cancer cell proliferation, invasion and apoptosis by modulating the Src/PI3K/Akt signaling pathway
Source: PeerJ. 2020 Mar 10;8:e8754. doi: 10.7717/peerj.8754 (PMC7069407; doi:10.7717/peerj.8754)

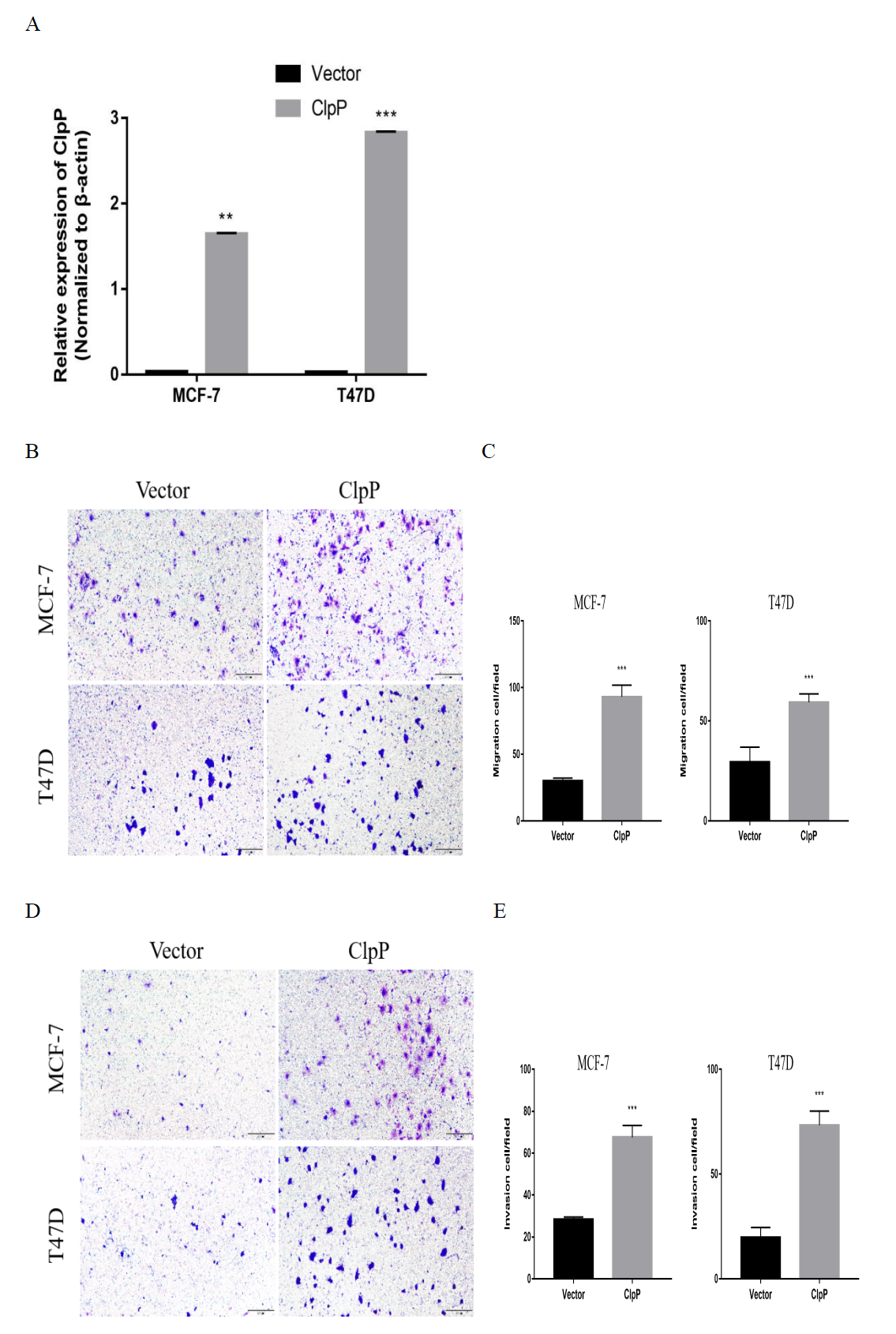

Supplement: Figure S1 — (A) The overexpressing efficiency in ClpP (MCF-7 and T47D cells) was evaluated by RT-qPCR. (B, C) The effects of overexpressed-ClpP on BC cell migration were evaluated using transwell assays. (D, E) The effects of overexpressed-ClpP on BC cell invasion were evaluated using transwell assays. The data are presented as mean ± SD, **p < 0.01, ***p < 0.001. [file peerj-08-8754-s001.png]

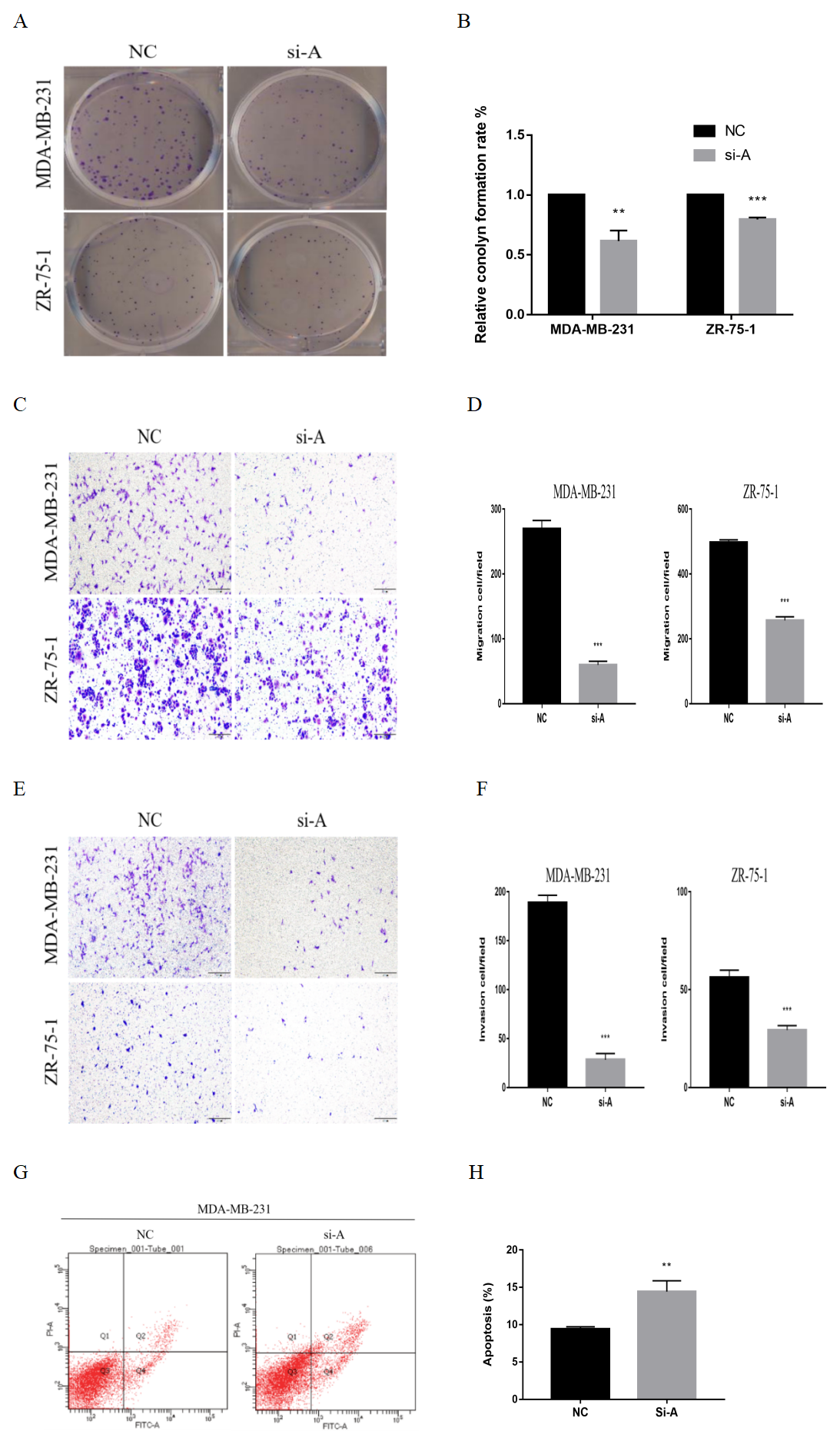

Supplement: Figure S2 — (A, B) The effects of si-ClpP-A on BC cell proliferation were analyzed using the colony formation assay. (C, D) The effects of si-ClpP-A on BC cell migration were evaluated using the transwell assay. (E, F) The effects of si-ClpP-A on BC cell invasion were evaluated using the transwell assay. (G, H) The effect of si-ClpP-A on MDA-MB-231 cells apoptosis was analyzed using flow cytometry. The data are presented as the mean ± SD, **p < 0.01, ***p < 0.001. [file peerj-08-8754-s002.png]

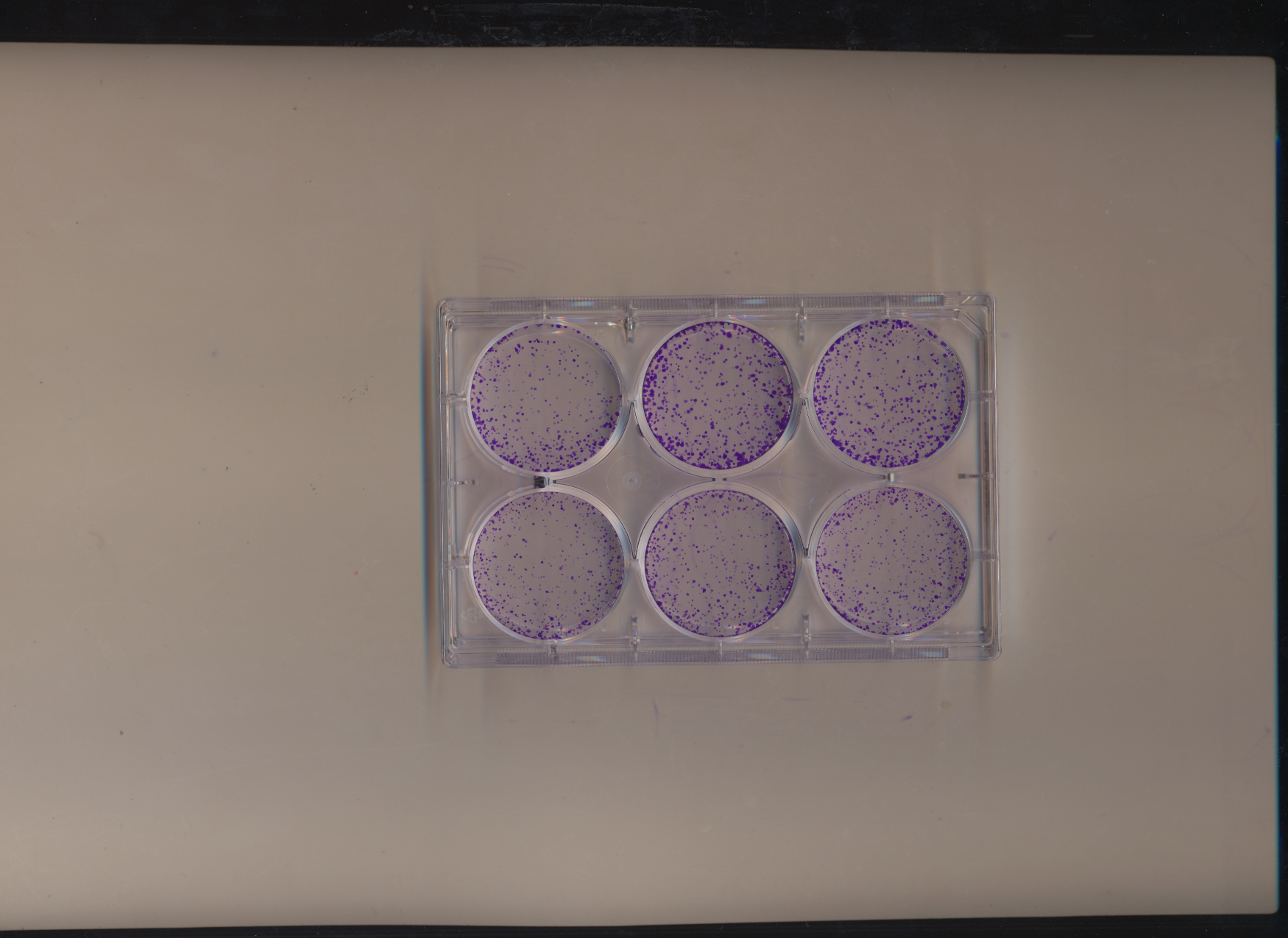

Supplement: Supplemental Information 40 [file peerj-08-8754-s040.tif]

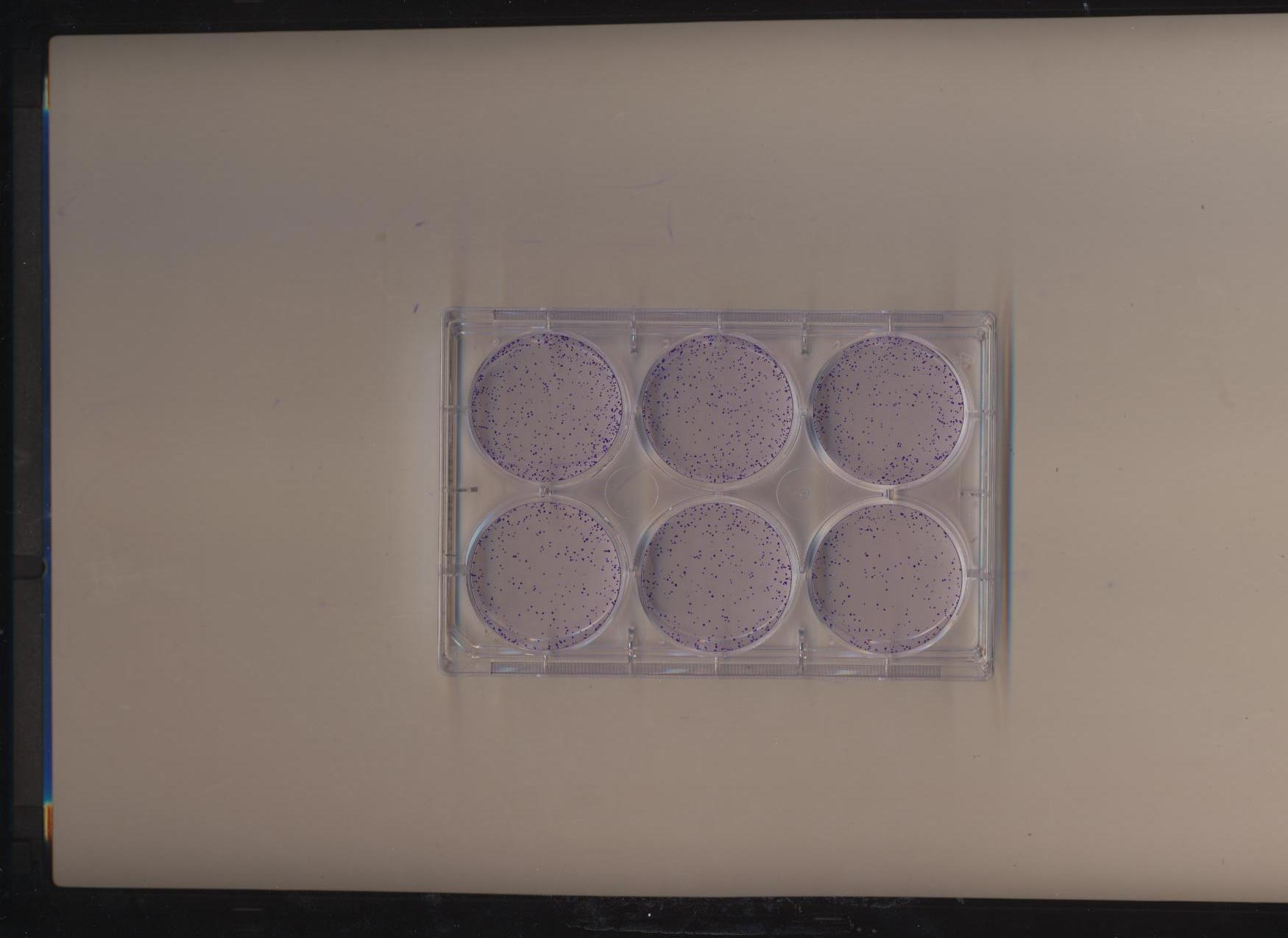

Supplement: Supplemental Information 41 [file peerj-08-8754-s041.jpg]

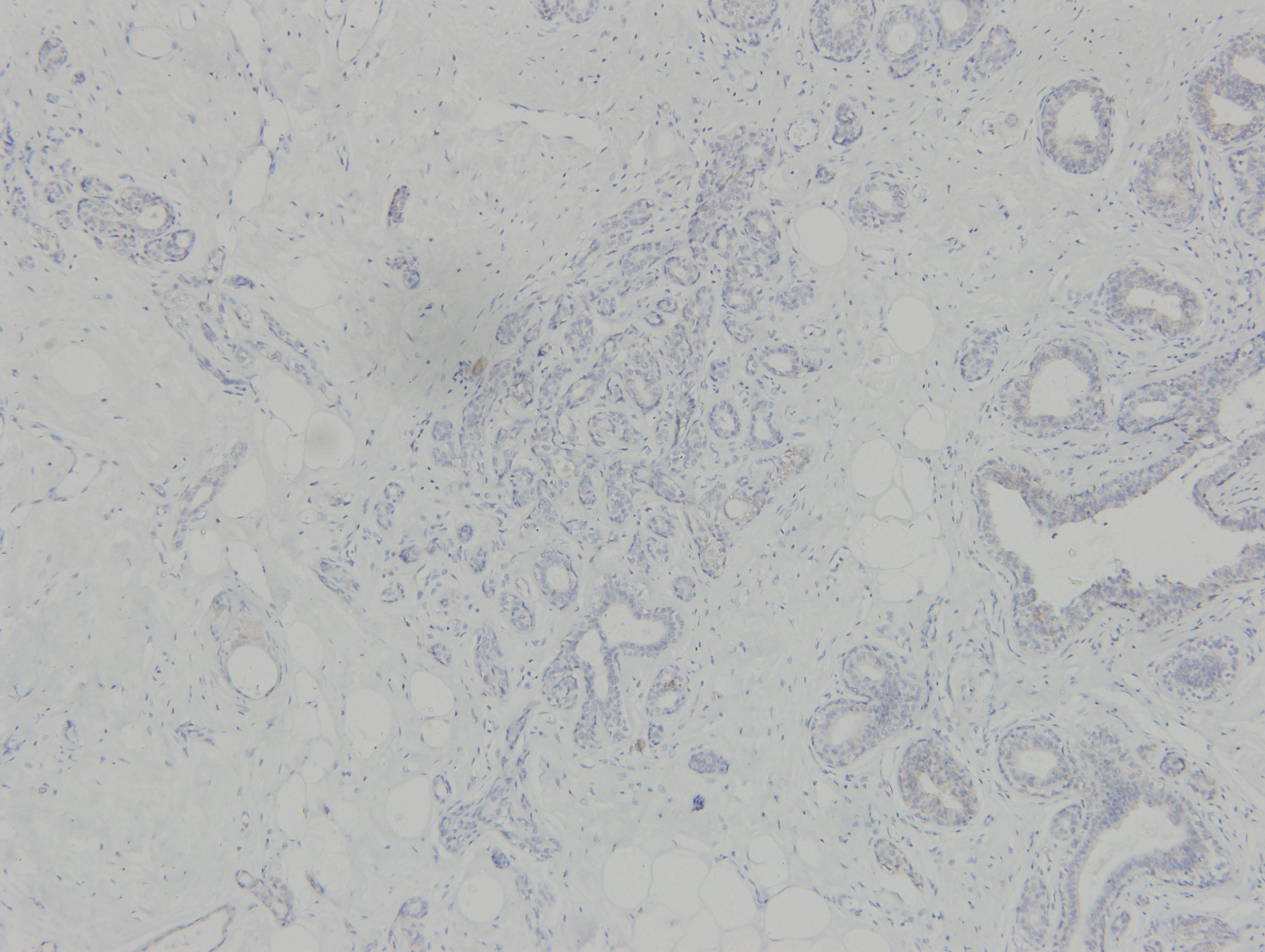

Supplement: Supplemental Information 44 [file peerj-08-8754-s044.jpg]

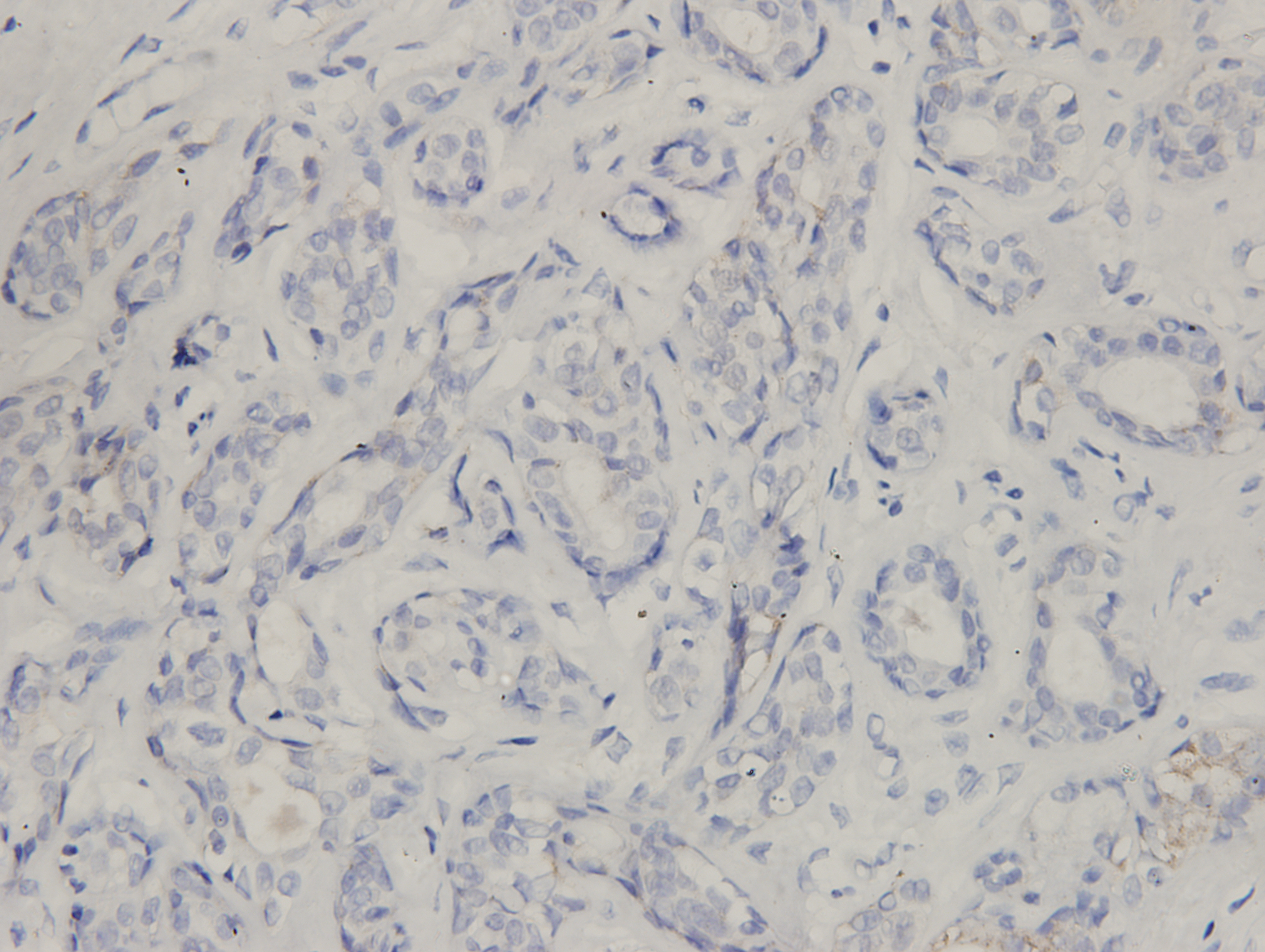

Supplement: Supplemental Information 45 [file peerj-08-8754-s045.jpg]

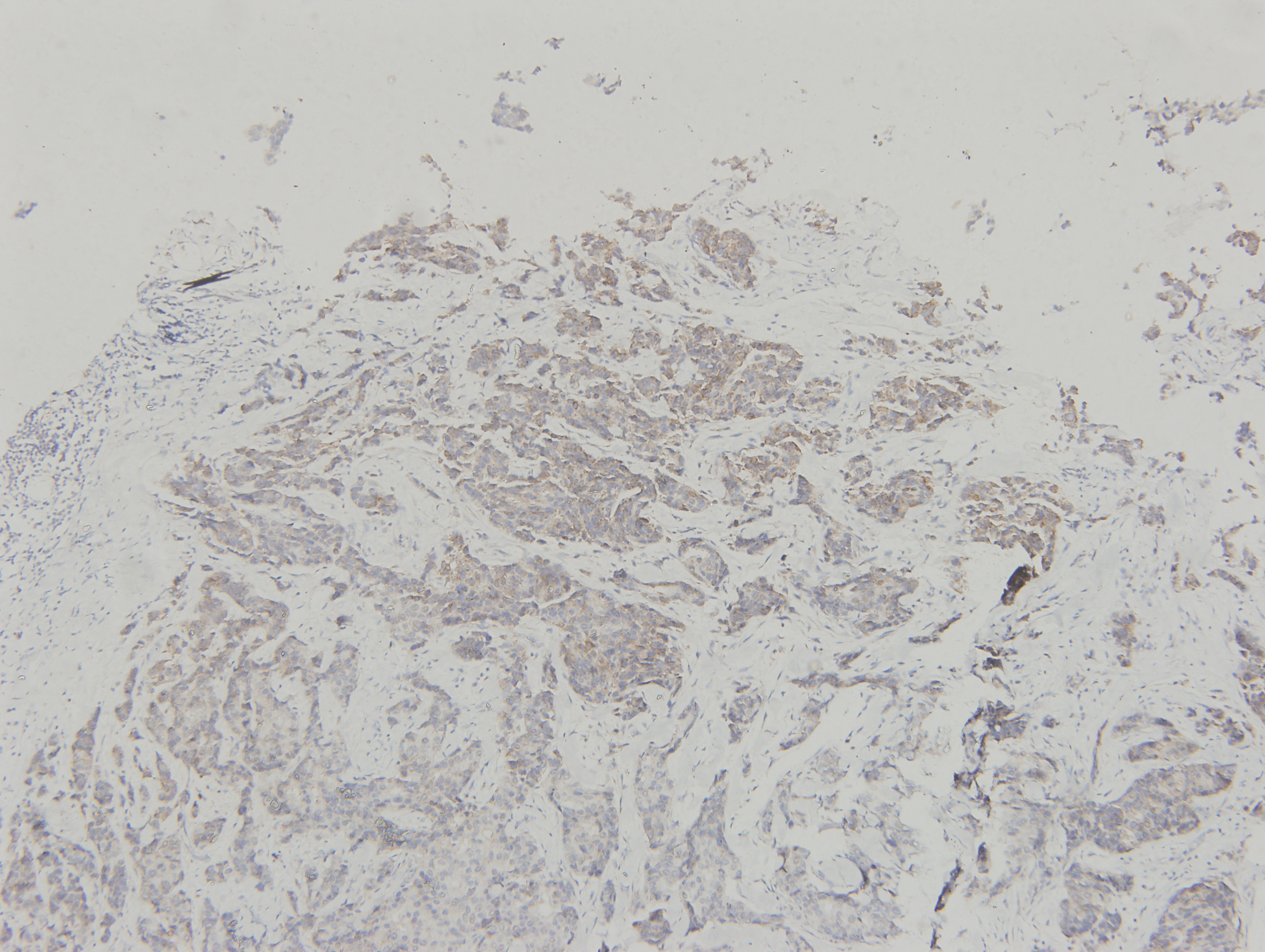

Supplement: Supplemental Information 46 [file peerj-08-8754-s046.jpg]

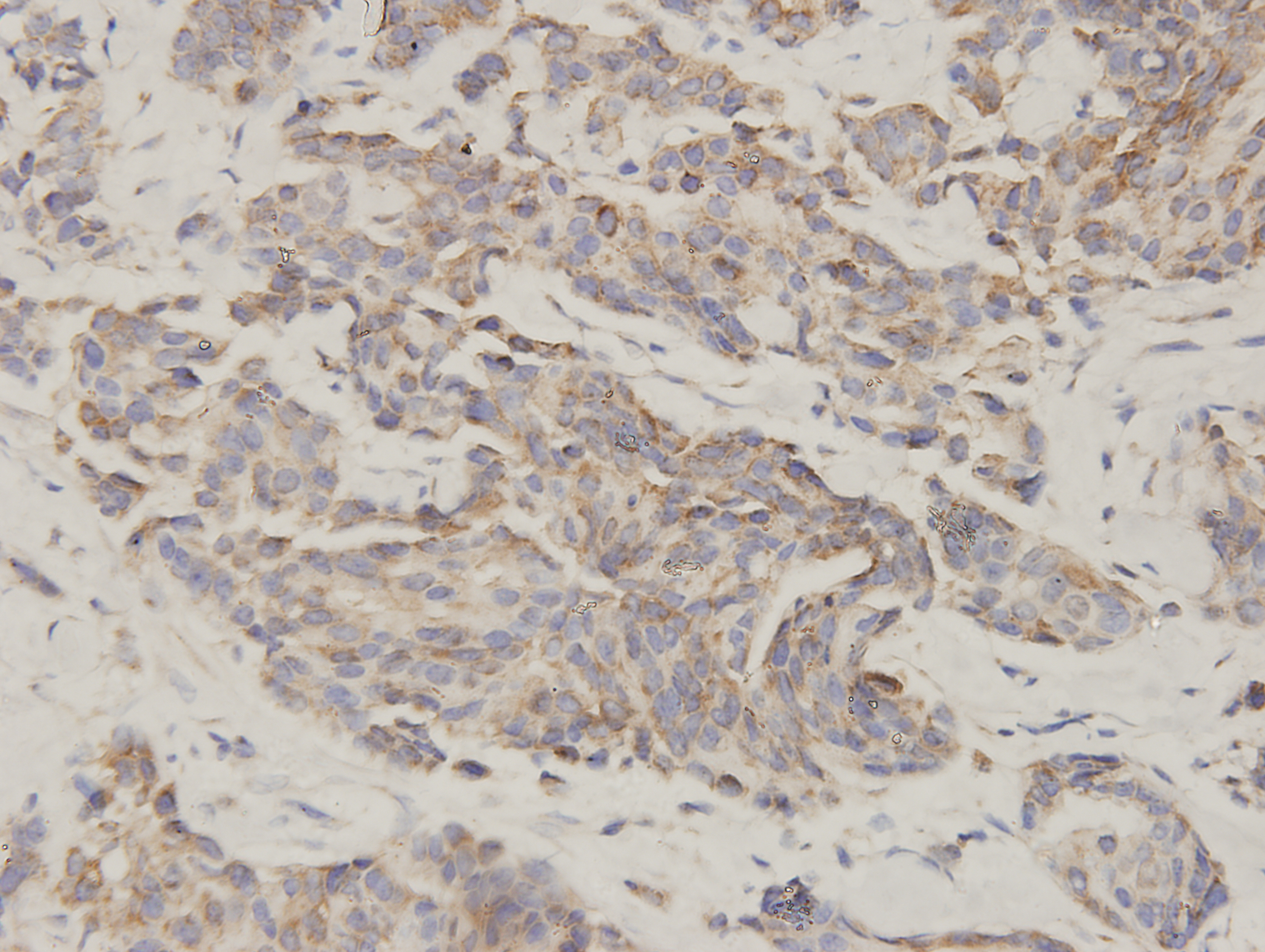

Supplement: Supplemental Information 47 [file peerj-08-8754-s047.jpg]
